# A Critical Review of Diagnostic Strategies and Maternal Offspring Complications in Gestational Diabetes Mellitus

**DOI:** 10.7759/cureus.51016

**Published:** 2023-12-24

**Authors:** Arya Thakur, Suyash Agrawal, Swarupa Chakole, Bhushan Wandile

**Affiliations:** 1 Medicine, Jawaharlal Nehru Medical College, Datta Meghe Institute of Higher Education and Research, Wardha, IND; 2 Community Medicine, Jawaharlal Nehru Medical College, Datta Meghe Institute of Higher Education and Research, Wardha, IND; 3 Nursing, Jawaharlal Nehru Medical College, Datta Meghe Institute of Higher Education and Research, Wardha, IND

**Keywords:** personalized care, early detection, diagnostic strategies, offspring complications, maternal complications, gestational diabetes mellitus (gdm)

## Abstract

Gestational diabetes mellitus (GDM) is a complex and significant health concern affecting pregnant individuals and their offspring. This review provides a comprehensive examination of GDM, focusing on diagnostic strategies and the associated maternal and offspring complications. We delve into the challenges and controversies surrounding GDM diagnosis, including the variability in diagnostic criteria, diagnostic accuracy and reproducibility issues, ethical considerations, and the influence of ethnicity and genetics. Maternal complications, such as preeclampsia, cesarean sections, long-term health implications, and neonatal complications like macrosomia, hypoglycemia, and respiratory distress syndrome, are explored in detail. Additionally, we investigate the long-term risks of childhood obesity and type 2 diabetes in offspring and potential cognitive and developmental outcomes. This review underscores the critical importance of early detection and effective management of GDM, the need for standardized diagnostic criteria, personalized care plans, and the ongoing pursuit of research to enhance our understanding of this complex condition. GDM remains a dynamic field where ongoing innovation and research promise to improve the health outcomes of pregnant individuals and their children.

## Introduction and background

Gestational diabetes mellitus (GDM) is a unique form of diabetes during pregnancy. It is characterized by elevated blood glucose levels that develop or are first recognized during pregnancy, typically during the second or third trimester. GDM is a significant health concern for both mothers and their offspring [1-3]. The pathophysiology of GDM involves insulin resistance, where the pregnant woman's body does not use insulin effectively, leading to increased blood sugar levels. This condition can have important implications for maternal and fetal health, making it a subject of substantial clinical and research interest [1].

GDM poses several risks to maternal health. High blood sugar levels in pregnant women with GDM can lead to complications such as preeclampsia, gestational hypertension, and an increased likelihood of requiring a cesarean section. Additionally, women with GDM have an elevated risk of developing type 2 diabetes later in life, emphasizing the importance of early diagnosis and management [4]. The impact on offspring health is equally concerning. Infants born to mothers with GDM are at risk of several complications, including macrosomia (excessive birth weight), hypoglycemia (low blood sugar), and respiratory distress syndrome. Furthermore, growing evidence suggests that exposure to GDM during pregnancy may increase the child's long-term risk of obesity and type 2 diabetes [5].

The primary objective of this review is to critically examine the diagnostic strategies employed in identifying GDM during pregnancy. It aims to assess the effectiveness, accuracy, and clinical implications of various diagnostic criteria, including the International Association of Diabetes and Pregnancy Study Groups (IADPSG) criteria, the American College of Obstetricians and Gynecologists (ACOG) criteria, fasting plasma glucose (FPG) testing, oral glucose tolerance tests (OGTT), hemoglobin A1c (HbA1c) as a potential diagnostic marker, and the use of continuous glucose monitoring (CGM) in GDM diagnosis.

## Review

Diagnostic Strategies for GDM

Overview of Current Diagnostic Criteria

Current diagnostic criteria for gestational diabetes mellitus (GDM) consist of two primary approaches different organizations recommend. The first approach, proposed by the International Association of Diabetes and Pregnancy Study Groups (IADPSG), employs a one-step method. Under these guidelines, pregnant individuals typically undergo a 75-gram oral glucose tolerance test (OGTT) between 24-28 weeks of gestation. Diagnosis relies on specific thresholds for fasting plasma glucose (FPG), one-hour glucose, and two-hour glucose levels during the test [6]. On the other hand, the American College of Obstetricians and Gynecologists (ACOG) advocates a two-step approach to GDM diagnosis. Initially, pregnant women are subjected to a 50-gram glucose challenge test (GCT) during the same gestational period. Suppose the GCT results indicate abnormal glucose levels (usually defined by specific thresholds); the individual proceeds to a more comprehensive 100-gram OGTT. The diagnosis is determined based on fasting glucose, one-hour, two-hour, and three-hour glucose values obtained during the OGTT [7]. These two diagnostic strategies reflect the ongoing debate surrounding GDM diagnosis, with the IADPSG approach favoring a single-step process and the ACOG approach utilizing a two-step method. The choice between these approaches often depends on regional practices and healthcare provider preferences, highlighting the need for further research to establish a standardized diagnostic criterion for GDM [8].

Fasting Plasma Glucose (FPG) Testing

Fasting plasma glucose (FPG) testing represents a straightforward and cost-effective diagnostic method for gestational diabetes mellitus. It offers advantages and disadvantages in the context of GDM diagnosis [9]. One of the significant advantages of FPG testing is its simplicity and convenience for patients. This diagnostic approach requires individuals to fast overnight before undergoing a blood glucose test, which is relatively straightforward and less burdensome than the oral glucose tolerance test. Additionally, FPG testing is less time-consuming, making it a practical option for pregnant individuals [10].

However, FPG testing has some limitations. One of its main drawbacks is its limited sensitivity, meaning it may miss some cases of GDM. This reduced sensitivity could lead to false negatives, where individuals with GDM may not receive a timely diagnosis. Moreover, FPG testing primarily measures fasting glucose levels. It does not capture postprandial (after-meal) glucose excursions, essential in GDM diagnosis, as they reflect the body's ability to handle glucose after consuming carbohydrates. The inability to assess postprandial glucose levels can be a significant limitation when diagnosing GDM, as it may not fully reflect the dynamic glucose changes during pregnancy. Consequently, FPG testing, while convenient, may not provide a comprehensive picture of glucose metabolism during pregnancy and may need to be complemented by other diagnostic methods to ensure accurate GDM diagnosis [11].

Oral Glucose Tolerance Test (OGTT)

The oral glucose tolerance test is a diagnostic method that offers a comprehensive assessment of glucose metabolism, providing a detailed evaluation of an individual's ability to handle glucose during and after consuming a glucose solution. Like other diagnostic approaches, the OGTT has advantages and disadvantages [12]. One of the significant advantages of the OGTT is its ability to offer a comprehensive overview of glucose tolerance. Unlike fasting plasma glucose FPG testing, the OGTT captures both fasting and postprandial (after-meal) hyperglycemia, which is essential in the diagnosis of GDM. This makes it a valuable tool for identifying glucose abnormalities that may not be evident through FPG testing alone [13].

However, the OGTT also comes with certain drawbacks. It is more time-consuming for patients, as it requires an overnight fast and the consumption of a glucose solution, followed by multiple blood draws at specified time intervals (typically at fasting, one-hour, two-hour, and sometimes three-hour intervals). This extended testing process can be burdensome for pregnant individuals, particularly when considering the discomfort of multiple blood draws [14].

Haemoglobin A1c (HbA1c) as a Potential Diagnostic Marker

The potential use of Hemoglobin A1c (HbA1c) as a diagnostic marker for gestational diabetes mellitus is a topic of interest, with both advantages and limitations to consider [15]. HbA1c is a measurement that reflects average blood glucose levels over the preceding 2-3 months, offering a view of overall glycemic control. One of the primary advantages of using HbA1c for GDM diagnosis is that it is a non-fasting test, which may enhance patient compliance and convenience. Unlike FPG or the OGTT, HbA1c does not require an overnight fast or the ingestion of glucose solutions, making it a more straightforward and accessible option for pregnant individuals [16].

However, there are notable limitations associated with using HbA1c for GDM diagnosis. Firstly, there needs to be more data available regarding its accuracy in diagnosing GDM, especially when compared to established diagnostic methods like the OGTT. GDM is a unique condition influenced by the physiological changes of pregnancy, which may impact the relationship between HbA1c levels and glucose control. Additionally, HbA1c levels can be influenced by various factors, including iron deficiency anemia and hemoglobinopathies, which may lead to inaccuracies in GDM diagnosis. Moreover, there is known variability in HbA1c levels during pregnancy, further complicating its use as a diagnostic marker [15]. Table 1 describes the diagnostic criteria for various tests used to diagnose GDM, along with their advantages and disadvantages.

**Table 1:** Outline of the diagnostic criteria for various tests used in the diagnosis of GDM, along with their advantages and disadvantages OGTT - oral glucose tolerance test; GCT - glucose challenge test; IADPSG - International Association of Diabetes and Pregnancy Study Group; ACOG - American College of Obstetricians and Gynecologists; GDM - gestational diabetes mellitus; FPG - fasting plasma glucose; HbA1c - hemoglobin A1c; CGM - continuous glucose monitoring

Diagnostic test	Diagnostic criteria	Advantages	Disadvantages
Glucose challenge test (GCT)	Non-fasting screening test. They are typically performed between 24-28 weeks of gestation. If positive results are followed by a more definitive test like the OGTT.	Simplicity and speed in comparison to more extensive tests. Non-fasting, making it convenient for pregnant women.	Lack of specificity, leading to potential false-positive results. Requires confirmation with a more comprehensive test if positive.
Oral glucose tolerance test (OGTT)	International Association of Diabetes and Pregnancy Study Group (IADPSG): 75-gram glucose load, specific thresholds for FPG, one-hour and two-hour glucose. American College of Obstetricians and Gynecologists (ACOG): 50-gram GCT followed by 100-gram OGTT with specific thresholds.	The gold standard for GDM diagnosis provides a comprehensive assessment. Can identify various degrees of glucose intolerance.	Fasting requirements may be less convenient for some pregnant women. It is time-consuming and involves multiple blood draws, causing discomfort. Variability in diagnostic criteria between organizations may lead to inconsistencies.
Fasting plasma glucose (FPG) Testing	Measures fasting glucose levels after overnight fasting.	Simplicity and convenience for patients. Less time-consuming compared to the OGTT.	Due to limited sensitivity, we may miss some cases of GDM (false negatives). It does not assess postprandial glucose excursions, which are potentially incomplete in reflecting dynamic glucose changes during pregnancy. It may need complementation with other tests.
Hemoglobin A1c (HbA1c)	Measures average blood glucose levels over the preceding two to three months.	Non-fasting test, enhancing patient compliance and convenience. Reflects overall glycemic control.	Limited data on accuracy for GDM diagnosis. It may not capture short-term variations in blood sugar levels. It is influenced by factors like iron deficiency anemia, impacting accuracy. Variability in HbA1c levels during pregnancy.
Continuous glucose monitoring (CGM)	Involves monitoring interstitial glucose levels over several days.	Offers real-time data on glucose fluctuations, capturing day-to-day variability. Provides comprehensive insights into glucose control.	More invasive compared to traditional tests. Higher cost and limited accessibility for some individuals. Requires specialized training for patients and healthcare providers. Interpretation of data requires expertise.

Continuous Glucose Monitoring (CGM) in GDM Diagnosis

Continuous glucose monitoring (CGM) has emerged as a promising approach in the context of GDM diagnosis, offering unique advantages and facing particular challenges [17]. CGM monitors interstitial glucose levels over several days, providing real-time data on glucose fluctuations throughout a patient's daily life. One of the significant advantages of CGM is its ability to offer a comprehensive view of glucose variability, capturing day-to-day fluctuations that traditional diagnostic tests may miss. This real-time data can provide valuable insights into an individual's glucose control, helping healthcare providers make more informed diagnostic decisions and tailor treatment plans [18].

However, there are some notable limitations associated with CGM when considering its use in GDM diagnosis. CGM is more invasive than traditional tests like fasting plasma glucose or the oral glucose tolerance test. It involves the insertion of a sensor beneath the skin, which may be uncomfortable or undesirable for some patients. Additionally, CGM technology is not universally available, and its higher cost may limit its accessibility for some individuals. Successful CGM use also requires specialized training for patients and healthcare providers and expertise in interpreting the collected data [19].

Pros and Cons of Each Diagnostic Method

GDM occurs during pregnancy and is characterized by elevated blood sugar levels. Various diagnostic methods are employed to identify GDM, each with its advantages and disadvantages [6]. The glucose challenge test (GCT) is a non-fasting screening test that offers simplicity and speed. It is convenient for pregnant women but may lack specificity, potentially leading to false-positive results. A positive GCT necessitates confirmation with a more definitive test, such as the OGTT [20]. The OGTT is the gold standard for GDM diagnosis due to its high accuracy. However, it requires fasting, making it less convenient for some pregnant women. The test is time-consuming and may cause discomfort due to consuming a larger glucose solution. Despite these drawbacks, OGTT is valuable for identifying various degrees of glucose intolerance [20]. HbA1c is an alternative method that reflects average blood sugar levels over a few months. It does not require fasting and offers convenience, but its application in pregnancy is not as well-established as OGTT. HbA1c may not capture short-term variations in blood sugar levels and may be affected by conditions impacting hemoglobin levels [6]. Continuous glucose monitoring provides real-time data and pattern recognition, offering insights into glucose variability. However, it is an invasive method involving sensor insertion, potentially causing discomfort. Cost is a significant consideration, and its role in GDM diagnosis is still under investigation [20].

Maternal complications of GDM

Hyperglycemia-Related Risks

Preeclampsia: Preeclampsia is a severe complication of pregnancy characterized by high blood pressure and damage to organs such as the liver and kidneys. Women with GDM are at an increased risk of developing preeclampsia compared to those without GDM. The exact mechanisms linking GDM to preeclampsia are complex and not fully understood, but they likely involve insulin resistance, inflammation, and endothelial dysfunction. Preeclampsia can lead to severe complications for both the mother and the fetus, making it a significant concern in GDM pregnancies [21-22].

Gestational hypertension: Gestational hypertension, characterized by elevated blood pressure during pregnancy without proteinuria (a key feature of preeclampsia), is more common in women with GDM. Like preeclampsia, the mechanisms linking GDM and gestational hypertension are not fully understood, but they likely involve shared risk factors such as insulin resistance and inflammation. Gestational hypertension can lead to adverse maternal and fetal outcomes and should be closely monitored in GDM pregnancies [23].

Long-Term Maternal Health Implications

Increased risk of type 2 diabetes: One of the significant concerns for women who have experienced GDM is the elevated risk of developing type 2 diabetes in the years following pregnancy. GDM is a precursor to type 2 diabetes, and women with GDM are at a higher risk of developing this chronic condition. This long-term health implication underscores the importance of postpartum monitoring and lifestyle modifications to reduce the risk of progression to type 2 diabetes [2].

Cardiovascular complications: GDM has been associated with an increased risk of cardiovascular disease (CVD) in the long term. Women with a history of GDM are more likely to develop hypertension, dyslipidemia, and coronary artery disease. Understanding the link between GDM and cardiovascular complications is crucial for developing strategies to mitigate these risks and improve the cardiovascular health of affected women [24]. GDM extends beyond hypertensive disorders, encompassing a spectrum of maternal and neonatal risks. While hypertensive disorders like preeclampsia are indeed significant complications associated with GDM, it's essential to recognize the broader range of potential health issues. Maternal complications include an elevated risk of cesarean sections, gestational hypertension, and long-term health implications such as an increased likelihood of developing type 2 diabetes later in life. Neonatal complications involve an enhanced risk of macrosomia (excessive birth weight), hypoglycemia (low blood sugar), and respiratory distress syndrome. Additionally, research indicates potential long-term risks for offspring, including a heightened susceptibility to childhood obesity and type 2 diabetes. By acknowledging the diverse array of complications associated with GDM, healthcare providers can implement more comprehensive monitoring and intervention strategies to optimize the health outcomes for both mothers and their children [24].

Impact on quality of life: GDM can substantially impact a woman's quality of life during pregnancy and beyond. The diagnosis and management of GDM often require significant lifestyle modifications, including dietary changes, increased physical activity, and glucose monitoring. These adjustments can be challenging and affect a woman's emotional well-being and overall quality of life. Additionally, long-term health concerns, such as the risk of type 2 diabetes and cardiovascular disease, can increase stress and anxiety [25].

Offspring Complications of GDM

Neonatal Complications

Macrosomia: Macrosomia, a condition characterized by newborns weighing significantly more than the average, typically exceeding 4,000 grams (8.8 pounds) at birth, poses a notable concern in maternal health. This phenomenon is particularly prevalent in infants born to mothers diagnosed with GDM. The heightened risk of macrosomia in these cases can be attributed to the exposure of the developing fetus to elevated levels of glucose in utero. The implications of macrosomia extend beyond its evident impact on birth weight, as it can lead to complications during childbirth. One such complication is shoulder dystocia, a condition in which the baby's head passes through the birth canal, but the shoulders become stuck behind the mother's pelvic bone. This obstetric emergency not only heightens the risk of birth injuries for the newborn but also poses potential dangers for the mother. The comprehensive understanding and management of macrosomia in pregnancies complicated by GDM are crucial for mitigating the associated risks and ensuring a safer childbirth experience for both mother and child [26].

Hypoglycemia: Hypoglycemia emerges as a prevalent complication in neonates born to mothers diagnosed with GDM. This condition manifests when the infant experiences a significant and abnormal decrease in blood sugar levels shortly after birth. The underlying mechanism is attributed to the infant's pancreas, which, exposed to elevated glucose levels in the uterine environment, may respond by producing an excess of insulin. This disproportionate insulin secretion can rapidly deplete the baby's blood glucose, leading to hypoglycemia. If left untreated, hypoglycemia in newborns poses serious risks, particularly in terms of neurological complications. Seizures and other adverse neurological effects are potential consequences, underscoring the critical importance of prompt and effective intervention. The management of neonatal hypoglycemia in infants born to mothers with GDM is essential to safeguard the neurological well-being of the newborn and ensure a healthy start to life [27].

Respiratory distress syndrome: Respiratory distress syndrome (RDS) is a challenging condition characterized by insufficiently developed lungs in newborns, impeding their ability to breathe effectively. This condition arises due to insufficient surfactant production, a substance essential for maintaining lung elasticity and preventing alveoli collapse. Infants born to mothers with GDM face a slightly elevated risk of RDS. This heightened susceptibility is associated with the complex interplay of factors potentially influenced by maternal health and the prenatal environment. The compromised lung development in these infants may result in breathing difficulties, necessitating specialized care in a neonatal intensive care unit (NICU). The NICU provides a controlled environment where newborns with RDS can receive medical attention, respiratory support, and monitoring to enhance their chances of overcoming respiratory challenges and achieving optimal lung function. Vigilant management and intervention are crucial in addressing RDS and ensuring the well-being of infants born to mothers with GDM [28].

Long-Term Health Risks in Offspring

Childhood obesity: The link between GDM during pregnancy and an elevated risk of childhood obesity in offspring has garnered attention, supported by accumulating evidence. While the precise mechanisms remain incompletely understood, it is believed that this association may be influenced by fetal overnutrition and the programming of adipose tissue development in utero. Exposure to elevated glucose levels during pregnancy may contribute to an altered metabolic environment for the developing fetus, potentially impacting long-term weight regulation. Childhood obesity, recognized as a substantial public health concern, brings with it a heightened risk of various health issues. Among these concerns are type 2 diabetes, cardiovascular disease, and metabolic syndrome, all of which underscore the far-reaching consequences of early-life factors on later health outcomes. The recognition of the potential connection between GDM and childhood obesity emphasizes the importance of proactive measures in maternal health and prenatal care to mitigate these long-term health risks for the offspring [29].

Type 2 diabetes risk: Exposure to GDM in utero has been associated with an increased risk of offspring developing type 2 diabetes later in life. This observation highlights the enduring impact of the intrauterine environment on a child's metabolic health. The mechanisms underlying this link may involve fetal exposure to elevated glucose levels, potentially influencing the development of metabolic pathways that contribute to a higher susceptibility to insulin resistance and impaired glucose regulation. Recognizing this association underscores the critical importance of early interventions aimed at mitigating the long-term risk of type 2 diabetes in individuals with a history of GDM exposure. Implementing lifestyle modifications, such as promoting healthy dietary habits and regular physical activity, becomes paramount. Additionally, regular monitoring and proactive management of metabolic health are crucial components of preventive strategies. By addressing these factors early in life, there is potential to reduce the risk of type 2 diabetes and promote overall long-term health in individuals who were exposed to GDM during their fetal development [30].

Cognitive and developmental outcomes: Research has illuminated potential cognitive and developmental implications for offspring born to mothers with GDM. Several studies have suggested a correlation between GDM and an elevated risk of neurodevelopmental disorders, including attention deficit hyperactivity disorder (ADHD) and autism spectrum disorders. The intricate interplay between maternal metabolic health during pregnancy and fetal neurodevelopment may contribute to these associations. Furthermore, investigations have indicated subtle cognitive differences in children exposed to GDM, potentially influencing academic performance and cognitive functioning. While the precise mechanisms linking GDM to these cognitive and developmental outcomes remain an area of ongoing study, the findings underscore the need for heightened awareness and monitoring of neurodevelopmental trajectories in children born to mothers with GDM. Early identification and intervention strategies may be crucial in addressing potential challenges and optimizing the cognitive and developmental well-being of these individuals [31].

Diagnostic challenges and controversies

Variability in Diagnostic Criteria

The diagnosis of GDM is complicated by the variability in diagnostic criteria used across different healthcare organizations and countries. For example, the International Association of Diabetes and Pregnancy Study Groups (IADPSG) criteria, the American College of Obstetricians and Gynecologists (ACOG) criteria, and other regional guidelines may have different thresholds for glucose levels and testing methods. This variability can lead to inconsistencies in identifying GDM cases, making it challenging to establish a universal standard for diagnosis [32].

Diagnostic Accuracy and Reproducibility

The accuracy and reproducibility of diagnostic tests for GDM are subject to ongoing debate. Different diagnostic methods have varying sensitivity and specificity, such as the oral glucose tolerance test (OGTT) and fasting plasma glucose (FPG) testing. Additionally, issues like laboratory variability and patient compliance can affect the reliability of test results. Ensuring consistent and accurate diagnosis is crucial to effectively manage GDM and reduce the risk of maternal and offspring complications [9].

Ethical Considerations in GDM Diagnosis

GDM diagnosis can raise ethical concerns, particularly related to the potential psychological impact on pregnant women. Receiving a GDM diagnosis can cause anxiety and stress, potentially leading to unnecessary medical interventions or lifestyle restrictions. Ethical considerations include disclosing GDM risk to pregnant individuals and the potential stigma associated with the condition. Balancing the benefits of early diagnosis with the psychological well-being of pregnant women is an essential ethical dilemma in GDM management [33].

The Role of Ethnicity and Genetics

Ethnicity and genetics play a significant role in the risk of developing GDM. Some populations, particularly those with a higher prevalence of type 2 diabetes, are at an increased risk for GDM. Additionally, genetic factors can influence an individual's susceptibility to GDM. Understanding the interplay between ethnicity, genetics, and GDM risk is crucial for tailoring diagnostic strategies and interventions to specific populations and individuals [34].

Emerging Trends in GDM Diagnosis

Advancements in medical technology and research continue to lead to emerging trends in GDM diagnosis. For example, CGM is gaining popularity as a diagnostic tool, providing real-time data on glucose levels. Additionally, research into novel biomarkers, such as hemoglobin A1c, may offer alternative diagnostic approaches. Evaluating the effectiveness and feasibility of these emerging trends in GDM diagnosis is essential to ensure the best possible care for pregnant individuals and their offspring [35].

Management and intervention

Lifestyle Modifications

Lifestyle modifications are fundamental to managing gestational diabetes mellitus. These adjustments are designed to help pregnant individuals control their blood sugar levels effectively and mitigate the risks associated with GDM. The core elements of lifestyle modifications encompass dietary changes, increased physical activity, and weight management, each playing a crucial role in promoting optimal health during pregnancy [36].

Dietary changes: Dietary modifications are pivotal in GDM management. Pregnant individuals with GDM are typically advised to adhere to a well-balanced, controlled carbohydrate diet. This dietary approach involves careful monitoring of carbohydrate intake, ensuring that it is distributed evenly throughout the day to prevent large spikes in blood glucose levels. Focusing on whole grains, fiber-rich foods, lean proteins, and healthy fats helps stabilize blood sugar levels. Additionally, avoiding high-sugar foods and sugary beverages is essential to prevent rapid fluctuations in glucose levels. Nutritional guidance and counseling by healthcare providers or registered dietitians are often integral in helping individuals make informed dietary choices tailored to their needs [37].

Physical activity: Regular physical activity is another key pillar of GDM management. Walking, swimming, or prenatal yoga can positively impact blood sugar control. Exercise improves insulin sensitivity, allowing the body to utilize glucose more effectively. It also helps maintain overall fitness and can contribute to a healthier pregnancy. Healthcare providers often work with individuals to establish an exercise routine that aligns with their fitness level, preferences, and potential contraindications [38].

Weight management: Maintaining a healthy weight during pregnancy is essential for individuals with GDM. Excessive weight gain can exacerbate insulin resistance and elevate the risk of complications. Healthcare providers typically recommend a targeted weight gain within a specific range based on the individual's pre-pregnancy body mass index (BMI). This personalized approach ensures weight management goals align with the patient's needs [39].

Pharmacological Interventions

While lifestyle modifications represent a cornerstone of managing GDM, there are situations where these adjustments alone may not adequately control blood sugar levels. Pharmacological interventions become crucial to GDM management [25]. These interventions aim to maintain glucose levels within a target range to reduce the risk of complications for both the mother and the baby. Common pharmacological options for GDM management include:

Insulin therapy is often the primary choice when lifestyle modifications alone do not achieve the desired blood sugar control. Insulin can be administered through subcutaneous injections or insulin pumps. It is considered safe for both the mother and the baby during pregnancy. Insulin allows for precise regulation of blood glucose levels and can be tailored to the individual's needs. Healthcare providers work closely with pregnant individuals to determine the appropriate insulin regimen, including the type of insulin, dosage, and timing of injections [40].

Oral antidiabetic medications in specific circumstances, mainly when insulin therapy is not preferred or feasible, may be considered an alternative to insulin. Metformin is one such medication that has been used for GDM management. However, its use in pregnancy requires careful monitoring and supervision by healthcare providers. The decision to prescribe oral medications over insulin is made on a case-by-case basis, considering the patient's medical history, preferences, and overall management plan [41].

Pharmacological interventions in GDM are tailored to individual needs and aim to achieve tight glycemic control while minimizing risks. Healthcare providers are pivotal in assessing the patient's circumstances and selecting the most suitable pharmacological approach. Close monitoring and regular follow-up appointments are essential to adjust medication regimens as needed and ensure that blood sugar levels remain within the target range throughout pregnancy. Ultimately, pharmacological interventions in GDM aim to optimize maternal and fetal outcomes while prioritizing safety and well-being [42].

Monitoring and Followup of GDM Cases

Effective monitoring and follow-up are paramount in managing GDM, ensuring the well-being of both the mother and the baby throughout the pregnancy [43]. These components of GDM care are crucial in tracking progress, adjusting treatment plans, and mitigating potential complications. Critical aspects of monitoring and follow-up include:

Blood glucose monitoring: Women diagnosed with GDM must monitor their blood glucose levels regularly. This involves self-administering blood glucose tests multiple times a day using a glucometer. Monitoring helps individuals and healthcare providers assess the impact of dietary modifications, physical activity, and, if necessary, pharmacological interventions on blood sugar control. Patients can make real-time adjustments to maintain glycemic targets by tracking glucose levels [44].

Prenatal care: GDM pregnancies necessitate close and vigilant prenatal care. Healthcare providers schedule regular prenatal check-ups to monitor both the mother's and the baby's health. These appointments include physical examinations, blood glucose monitoring discussions, and maternal and fetal well-being assessments. Ultrasounds are often employed to monitor fetal growth and assess for any potential complications, such as macrosomia [45].

Glycemic control assessment: Regular assessments of glycemic control are fundamental in GDM management. Healthcare providers analyze the data from blood glucose monitoring to evaluate how well the treatment plan works. Based on these assessments, adjustments to treatment may be recommended, such as modifying medication doses, altering dietary recommendations, or modifying the exercise regimen. The goal is to maintain blood glucose levels within the target range to minimize risks to the mother and the baby [46].

Shared Decision-Making Between Healthcare Providers and Patients

Shared decision-making is a vital and patient-centered approach to managing GDM. It fosters open and collaborative communication between healthcare providers and pregnant individuals, ensuring that treatment plans align with individual needs and preferences [47]. The key principles of shared decision-making in GDM management include:

Informed consent: Healthcare providers are crucial in facilitating informed consent. They should provide comprehensive information about available treatment options, potential risks, benefits, and possible outcomes associated with each choice. This empowers pregnant individuals to make well-informed decisions about their care, considering their unique circumstances and preferences. Informed consent is essential when discussing interventions like pharmacological treatments or the mode of delivery [48].

Patient education: Education is a cornerstone of shared decision-making. Pregnant individuals should receive thorough and accessible education about GDM, its management strategies, and the significance of adhering to treatment plans. Education empowers patients to participate in their care actively, make informed choices, and understand the implications of their decisions. It includes guidance on dietary modifications, blood glucose monitoring, and the importance of physical activity [49].

Individualized care plans: GDM management should be personalized to each patient's needs and circumstances. When developing treatment plans, healthcare providers should consider age, gestational age, medical history, and personal preferences. Tailoring care to the individual ensures appropriate and effective interventions, enhancing patient adherence and satisfaction [50].

Emotional support: GDM can present emotional challenges for pregnant individuals, including anxiety, stress, and concerns about the health of both the mother and the baby. Healthcare providers should offer emotional support and resources to address these concerns. Open and empathetic communication can help individuals cope with the emotional aspects of GDM and foster a sense of reassurance and confidence in their care [51].

*Recent* A*dvances in Diagnosing GDM*

Recent advances in diagnosing GDM have introduced innovative approaches to improve accuracy, convenience, and patient outcomes. CGM has emerged as a promising tool, providing real-time data on glucose levels over an extended period. This technology provides a comprehensive understanding of glucose variability, offering insights into daily fluctuations that traditional tests might miss. Additionally, CGM can potentially enhance personalized care plans by tailoring interventions based on dynamic glucose patterns. Moreover, advancements in machine learning algorithms to analyze CGM data hold promise in refining diagnostic precision and predicting GDM risk more effectively. Furthermore, ongoing research explores the utility of novel biomarkers beyond traditional glucose-based measurements. Integrating these advancements into routine clinical practice could revolutionize GDM diagnosis, facilitating early detection, more accurate risk stratification, and tailored interventions for pregnant individuals at risk of GDM. As technology continues to evolve, combining these emerging diagnostic approaches may contribute to a more nuanced and effective management of GDM [50].

## Conclusions

In conclusion, GDM presents a multifaceted maternal and fetal health challenge. This critical review has illuminated the intricacies surrounding GDM, including the variability in diagnostic criteria, diagnostic accuracy, and the ethical considerations associated with its diagnosis. We have also explored the array of complications that GDM can bestow upon both mothers and their offspring, from preeclampsia to the long-term risks of type 2 diabetes in children. Early detection and effective management are paramount in mitigating these risks. Furthermore, the need for standardized diagnostic criteria and personalized care plans, considering ethnicity and genetics, must be balanced. As we look to the future, continued research efforts hold the promise of refining diagnostic approaches, identifying novel biomarkers, and ultimately improving the outcomes for pregnant individuals and their children. GDM remains a dynamic and evolving field, demanding ongoing attention and innovation to ensure the best possible health for mothers and their offspring.
